# Early Pregnancy Glycosylated Hemoglobin in Relation to Gestational Diabetes Mellitus and Adverse Pregnancy Outcomes: Evidence from a Multisite Low- and Middle-Income Countries Cohort

**DOI:** 10.64898/2026.09.23.26362980

**Published:** 2026-09-29

**Authors:** Wen-Chien Yang, Sarah Sunil Joseph, B Vijayalekshmi, Bethany L Freeman, Muhammad Imran Nisar, Qing Pan, Santosh Joseph Benjamin, Jayakumar Amirtharaj G, Neeraj Sharma, Sarmila Mazumder, Zahra Hoodbhoy, Victor Akelo, Florence Aweyo, Emily R Smith, Christopher N Mores, Erin M Oakley, M Bridget Spelke, Humphrey Mwape, Sarah Mukuka, Margaret P Kasaro, Anne George Cherian

## Abstract

**Background:**

Oral glucose tolerance test (OGTT), the gold standard for diagnosing gestational diabetes mellitus (GDM), faces significant implementation challenges, prompting interest in alternative biomarkers, such as glycated hemoglobin (HbA1c), for early detection. This study aimed to assess the association between early pregnancy HbA1c and subsequent GDM and adverse perinatal outcomes in low- and middle-income countries.

**Methods:**

We analyzed data from the **Pregnancy Risk, Infant Surveillance, and Measurement Alliance - Maternal and Newborn Health** (PRISMA-MNH) cohort, which followed pregnant women enrolled before 20 weeks of gestation across five study sites in Kenya, India, Pakistan, and Zambia. We excluded participants with overt diabetes, severe anemia (Hb <7.0 g/dl), multiple gestations, or pregnancies that ended before 24 weeks of gestation. HbA1c was measured at enrollment. The primary outcome was GDM, diagnosed according to the International Association of Diabetes and Pregnancy Study Groups (IADPSG) criteria based on OGTT performed after 24 weeks of gestation. Secondary outcomes included hypertensive disorder of pregnancy, emergent Cesarean section, preterm birth, large for gestational age, and stillbirth after 28 weeks. We used AUC and ROC analyses to evaluate predictive performance and single- and two-threshold approaches to identify optimal cutoffs for predicting GDM. We used modified Poisson regression with robust standard errors to assess associations between early HbA1c and outcomes, and meta-analysis to pool site-specific estimates.

**Results:**

Among 10,918 pregnancies, GDM prevalence was 6.4%. Women with GDM were older (28.0±5.4 versus 26.0±5.3 years) and had higher BMI at enrollment (25.0±5.8 versus 22.9±5.0 kg/m ^2^ ) than women without GDM. The AUC for HbA1c alone in predicting GDM was 0.65 (95% confidence interval 0.63–0.67). A single early HbA1c threshold of 5.4%, based on the largest Youden’s index, showed limited predictive performance (sensitivity 46.7%, specificity 75.8%). A two-threshold approach identified rule-out and rule-in cutoffs of 5.0% (sensitivity 80.3%, specificity 35.6%) and 5.5% (sensitivity 34.5%, specificity 83.1%), respectively. In adjusted models, each 0.1% increase in HbA1c was associated with a 12% higher risk (RR 1.12, 95% CI 1.10–1.15) of GDM, while no significant associations were observed for any secondary outcomes.

**Conclusion:**

Early pregnancy HbA1c is independently associated with an increased risk of subsequent GDM. However, it demonstrated limited discriminatory ability as a standalone alternative to OGTT for identifying women who would later develop GDM. The identified rule-out threshold may help identify women at low risk of GDM, although the majority would still require OGTT for definitive diagnosis.

## Full Text Availability

The license terms selected by the author(s) for this preprint version do not permit archiving in PMC. The full text is available from the preprint server.

